# Insomnia and Sleep Disturbance in Stroke Survivors: A Meta-Analysis of Prevalence, Risk Factors, and Recovery Outcomes in Ischemic and Hemorrhagic Stroke Survivors

**DOI:** 10.7759/cureus.113184

**Published:** 2026-07-22

**Authors:** Ndilmadji G Mogombaye, Dhierin Jagdewsing, Wally Elijah, Fatemeh Rashidi, Masoumeh Rashidi, Hania Moharam, Sima Jagdewsing, Angel, Fariya Iqbal, Limei Liu

**Affiliations:** 1 Department of Neurology, Dalian Medical University, Dalian, CHN; 2 Department of General Surgery, Dalian Medical University, Dalian, CHN; 3 Department of Anesthesiology, Sir Run Run Hospital of Nanjing Medical University, Nanjing, CHN; 4 Department of Clinical Medicine, Nanjing Medical University, Nanjing, CHN; 5 Department of Neurology, Curaçao Medical Center (CMC), Willemstad, CUW; 6 Department of Internal Medicine, Nanjing Medical University, Nanjing, CHN; 7 Department of Internal Medicine, Dalian Medical University, Dalian, CHN; 8 Department of Neurology, Second Affiliated Hospital of Dalian Medical University, Dalian, CHN

**Keywords:** hemorrhagic stroke, insomnia, ischemic stroke, sleep disturbance, stroke survivor

## Abstract

Post-stroke sleep disturbance/insomnia is a prevalent but under-recognized complication that impairs recovery and the quality of life in stroke survivors. This meta-analysis aims to determine the prevalence of sleep disturbance/insomnia after stroke, compare sleep disturbance/insomnia rates between ischemic and hemorrhagic stroke patients, evaluate demographic and regional influences, and assess the impact of comorbidities such as anxiety, depression, and cardiovascular conditions on post-stroke sleep disturbance/insomnia. This meta-analysis was registered with the International Prospective Register of Systematic Reviews (PROSPERO) (registration number: CRD42024622432). A systematic search was conducted across PubMed, Scopus, Web of Science, and the Cochrane Library to identify observational studies reporting sleep disturbance or insomnia among adult stroke survivors. Data on stroke subtypes, sleep disturbance/insomnia prevalence, risk factors, and diagnostic methods were extracted. Quality was assessed using the Newcastle-Ottawa Scale (NOS). Pooled odds ratios (ORs) with 95% confidence intervals (CIs) were calculated using a random-effects model. Subgroup analyses evaluated variations by sleep disturbance/insomnia definition, age, stroke type, follow-up duration, and region. Thirteen studies encompassing 6,109 stroke survivors were included. Women, patients with anxiety, depression, or heart disease, showed significantly higher odds of post-stroke sleep disturbance/insomnia. Subgroup analyses suggested that sleep disturbance/insomnia during later follow-up (>3 months) was more pronounced. Post-stroke sleep disturbance/insomnia is influenced by psychological and clinical factors, with heightened vulnerability in female patients, anxiety, depression, and existing heart conditions. These findings support the integration of sleep assessments and targeted interventions into stroke rehabilitation to enhance recovery outcomes.

## Introduction and background

Stroke is a neurological disorder caused by an interruption of blood flow to the brain, resulting in brain tissue injury and neurological dysfunction. Stroke can broadly be classified into ischemic stroke, caused by vascular occlusion, and hemorrhagic stroke, caused by intracranial bleeding. It is a leading cause of disability worldwide, often resulting in a range of physical, cognitive, and emotional challenges for survivors [1]. Among these challenges, sleep disturbance/insomnia is a prevalent yet frequently under-recognized complication [2]. Sleep disturbance/insomnia, characterized by difficulties maintaining good sleep, affects roughly 30%-37% of stroke survivors within the first year post-stroke, with a higher prevalence in women than men [3]. A systematic review and meta-analysis reported a prevalence of 38.2% for sleep disturbance/insomnia symptoms in individuals affected by stroke [4]. This prevalence suggests that stroke may disrupt normal sleep regulation through neurological and psychological mechanisms, contributing to impaired sleep quality, circadian rhythm disturbances, and insomnia symptoms [5,6]. These disturbances may negatively affect rehabilitation, recovery, and the quality of life in stroke survivors [3,7]. Moreover, stroke survivors experiencing chronic sleep disturbance/insomnia are less likely to return to work and exhibit poorer functional outcomes compared to those without sleep disturbance/insomnia [3]. Stroke-related damage to brain regions involved in sleep-wake regulation, such as the thalamus and brainstem, can directly influence sleep patterns.

Additionally, psychological factors, including post-stroke depression and anxiety, further contribute to the development of sleep disturbance/insomnia [5]. Addressing sleep disturbance/insomnia in stroke survivors is crucial, as untreated sleep disturbance/insomnia can prolong rehabilitation efforts and recovery [6,8]. The effective management of post-stroke sleep disturbances, including insomnia symptoms, impaired sleep quality, fragmented sleep, excessive daytime sleepiness, and circadian rhythm disturbances, may involve a combination of pharmacological and non-pharmacological interventions tailored to the individual's clinical condition and underlying contributing factors [5].

Post-stroke sleep disturbance/insomnia significantly affects recovery and the quality of life, yet research regarding its prevalence, associated risk factors, and differences between ischemic and hemorrhagic stroke survivors remains limited. This meta-analysis aimed to evaluate the prevalence of post-stroke sleep disturbance/insomnia, identify associated demographic and clinical risk factors, and compare findings between ischemic and hemorrhagic stroke survivors. We hypothesized that post-stroke sleep disturbance/insomnia would be associated with psychological and clinical comorbidities and may negatively influence recovery outcomes.

## Review

Research design

This study employed a systematic review and meta-analysis of observational studies to investigate the prevalence and associated factors of post-stroke insomnia and sleep disturbances among adult stroke survivors. The research was designed in accordance with Preferred Reporting Items for Systematic Reviews and Meta-Analyses (PRISMA) guidelines and focused on synthesizing data from cohort, case-control, and cross-sectional studies. Randomized controlled trials were excluded because this study aimed to evaluate the prevalence and risk factors of post-stroke sleep disturbance/insomnia rather than treatment effects. Therefore, observational studies were considered more appropriate for the research question.

Inclusion and exclusion criteria

This study included adult stroke survivors (≥18 years) with hemorrhagic or ischemic strokes, at various recovery stages, to analyze post-stroke sleep disturbance/insomnia and its factors. Only studies that measured insomnia or sleep disturbance/insomnia as a primary or secondary outcome were included. Randomized controlled trials were excluded because this study focused on the prevalence and risk factors of post-stroke sleep disturbance/insomnia rather than treatment interventions. Therefore, only observational study designs, including cohort, case-control, and cross-sectional studies, were considered. Studies were included if they provided sufficient quantitative data on post-stroke sleep disturbance/insomnia prevalence or associated risk factors, including studies comparing stroke subtypes or patients with and without sleep disturbance/insomnia. Only studies published in English were included.

Data sources and search strategy

A systematic search was conducted across PubMed, Cochrane Library, Scopus, and Web of Science, covering medical, neurological, and sleep-related research. Additional studies were identified through the manual searching of reference lists and citation tracking to ensure a comprehensive data collection process. A structured search strategy was employed using MeSH terms and keywords such as "stroke," "sleep disturbance," "insomnia," and "sleep disorders." Filters for human studies and English-language publications were applied. No publication date restrictions were used, ensuring that both historical and recent studies were included.

Data extraction

Data were extracted using predefined study variables, including study characteristics, stroke type, sleep disturbance/insomnia assessment methods, and outcome measures such as prevalence and associated risk factors. Odds ratios (ORs) were manually calculated from available event and non-event data. Two reviewers independently extracted data, resolving discrepancies through discussion to ensure accuracy.

Quality assessment

The Newcastle-Ottawa Scale (NOS) was used to assess the quality of observational studies, focusing on selection, comparability, and outcome measurement [9]. Studies were categorized as low, moderate, or high risk of bias. Heterogeneity was assessed using the I² statistic, and subgroup analyses were conducted to identify potential sources of variability. Two authors independently rated each study, and discrepancies were resolved through discussion to reach consensus.

Statistical analysis

All statistical analyses were conducted using R software version 4.4.3 (R Foundation for Statistical Computing, Vienna, Austria) with the meta and metafor packages. Prevalence estimates and pooled proportions with 95% confidence intervals (CIs) were calculated from extracted event and total sample data using random-effects proportion-based analyses. Comparative binary outcomes were analyzed using random-effects meta-analysis models to calculate pooled odds ratios (ORs) with 95% CIs. The metabin() function was used for comparative binary analyses of extracted event and non-event data. Between-study heterogeneity was assessed using Cochran's Q test and the I² statistic, with DerSimonian-Laird estimation applied for between-study variance (τ²). Forest plots were generated to visualize pooled estimates and subgroup analyses, while funnel plots and Egger's test were used to assess publication bias where applicable. A p-value of <0.05 was considered statistically significant.

Result

**Figure 1:**
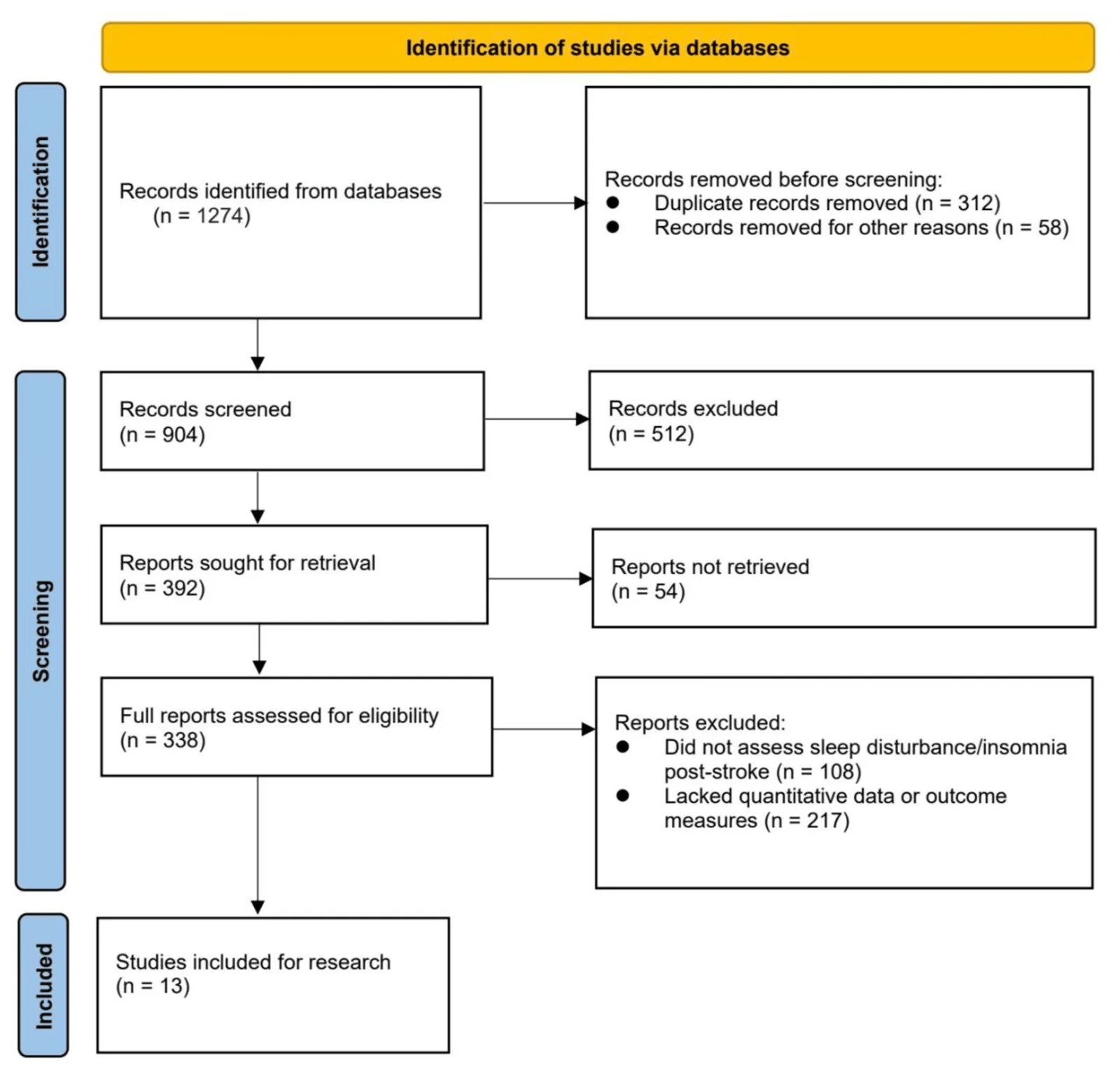
PRISMA flowchart illustrating study selection for the meta-analysis of post-stroke sleep disturbance/insomnia PRISMA: Preferred Reporting Items for Systematic Reviews and Meta-Analyses

Figure 1 illustrates the PRISMA flow diagram of the systematic study selection process conducted for the meta-analysis on post-stroke sleep disturbance/insomnia in stroke survivors. A total of 1,274 records were identified through database searches. After removing 312 duplicate records and 58 records for other reasons (e.g., non-English articles and abstract-only publications), 904 records remained for screening based on title and abstract. Following initial screening, 512 records were excluded for irrelevance. Of the 392 full-text reports sought, 54 could not be retrieved due to access limitations. The remaining 338 full-text articles were assessed for eligibility. Among these, 108 studies did not specifically assess sleep disturbance/insomnia post-stroke, and 217 lacked quantitative data or usable outcome measures. Ultimately, 13 studies met the eligibility criteria and were included in the final meta-analysis.

As shown in Table 1, this meta-analysis included 13 observational studies conducted across multiple countries, including China, Finland, South Korea, Australia, Hong Kong, and Taiwan. These studies evaluated post-stroke sleep disturbance or insomnia among adult ischemic and/or hemorrhagic stroke survivors, employing diverse assessment tools such as the Diagnostic and Statistical Manual of Mental Disorders, Fourth Edition (DSM-4); DSM, Fifth Edition (DSM-5); Pittsburgh Sleep Quality Index (PSQI); Athens Insomnia Scale (AIS); Insomnia Severity Index (ISI); and self-reported measures. Most studies were prospective cohorts, with a smaller proportion being retrospective in design. The variables analyzed in each study are detailed in Table 2, with the frequent inclusion of demographic (e.g., age, sex, and marital status), behavioral (e.g., smoking and alcohol use), and clinical factors (e.g., hypertension, diabetes, anxiety, and depression). Study quality, assessed using the Newcastle-Ottawa Scale (NOS), is summarized in Table 3, where seven studies scored 9 points and were rated as high quality, while six studies scored 6 points and were classified as moderate quality. Prevalence estimates of sleep disturbance/insomnia are presented in Table 4, ranging widely from 15.6% to 81.2%, which likely reflects methodological and population heterogeneity across studies. These findings emphasize the variability in measurement and prevalence, supporting the need for standardization in future insomnia research among stroke populations.

**Table 1:** Included studies and their information PSQI, Pittsburgh Sleep Quality Index; ISI, Insomnia Severity Index; AIS, Athens Insomnia Scale; DSM-4, Diagnostic and Statistical Manual of Mental Disorders, Fourth Edition; DSM-5, Diagnostic and Statistical Manual of Mental Disorders, Fifth Edition

Study ID	Stroke Type	Study Design	Stroke Type	Total Sample Size	Sleep Disturbance/Insomnia	Sleep Assessment Tool/Definition
Leppävuori et al., 2002 [10]	Finland	Prospective cohort	Ischemic	277	157	DSM-4 criteria
Fan et al., 2022 [11]	China	Prospective cohort	Ischemic	1,401	1,137	Difficulty falling asleep, staying asleep, or waking early
Moon et al., 2018 [12]	South Korea	Retrospective cohort	Ischemic and hemorrhagic	140	35	PSQI ≥ 5
Li et al., 2018 [13]	China	Retrospective cohort	Ischemic and hemorrhagic	1,062	489	Karolinska Sleep Questionnaire
Iddagoda et al., 2020 [7]	Australia	Prospective cohort	Ischemic and hemorrhagic	104	31	PSQI ≥ 9, ISI ≥ 15, and AIS ≥ 8
Niu et al., 2023 [14]	China	Prospective cohort	Ischemic	569	89	Frequent difficulty in initiating sleep, maintaining sleep, or early waking
Xiao et al., 2020 [15]	China	Prospective cohort	Ischemic	327	76	Athens Insomnia Scale score > 6
Chen et al., 2011 [16]	Hong Kong	Prospective cohort	Ischemic	508	186	Self-reported Athens Insomnia Scale score
He et al., 2019 [17]	China	Prospective cohort	Ischemic	152	24	PSQI ≥ 8
Xu et al., 2023 [18]	China	Prospective cohort	Ischemic	508	144	Self-reported insomnia
Niu et al., 2023 [19]	China	Retrospective cohort	Ischemic	530	311	DSM-5 + Athens Insomnia Scale
Tang et al., 2015 [20]	Hong Kong	Prospective cohort	Hemorrhagic	336	149	DSM-5
Tsai et al., 2022 [21]	Taiwan	Prospective cohort	Ischemic	195	58	Athens Insomnia Scale score > 6

**Table 2:** Variables analyzed in each included study

Study	Ischemic Stroke	Hemorrhagic Stroke	Sleep Disturbance/Insomnia	Age	Sex	Smoking	Drinking	Married	Work	Hypertension	Diabetes	Hyperlipidemia	Anxiety	Depression	BMI	Heart Conditions
Leppävuori et al., 2002 [10]	X		X	X	X	X	X	X			X		X	X		
Fan et al., 2022 [11]	X		X	X	X	X	X	X	X	X	X	X	X	X		
Moon et al., 2018 [12]	X	X	X	X	X											
Li et al., 2018 [13]	X	X	X	X	X	X		X	X	X	X	X		X		
Iddagoda et al., 2020 [7]	X	X	X	X	X											
Niu et al., 2023 [14]	X		X	X	X	X	X	X	X	X	X				X	X
Xiao et al., 2020 [15]	X		X	X	X	X	X	X		X	X		X			X
Chen et al., 2011 [16]	X		X	X	X	X	X	X		X	X	X				X
He et al., 2019 [17]	X		X	X	X	X				X	X	X				
Xu et al., 2023 [18]	X		X	X	X					X	X					X
Niu et al., 2023 [19]	X		X	X	X	X	X	X	X	X	X	X			X	
Tang et al., 2015 [20]		X	X	X	X					X	X					X
Tsai et al., 2022 [21]	X		X	X	X	X	X			X	X					X

**Table 3:** Quality assessment score (NOS) Studies were assessed using the Newcastle-Ottawa Scale (NOS), evaluating selection (0-4), comparability (0-2), and outcome/exposure (0-3), for a total score out of 9. Quality was categorized as high (7-9), moderate (4-6), or low (0-3)

Study	Selection (0-4)	Comparability (0-2)	Outcome/Exposure (0-3)	Total Score (0-9)	Quality
Leppävuori et al., 2002 [10]	4	2	3	9	High
Fan et al., 2022 [11]	3	1	2	6	Moderate
Moon et al., 2018 [12]	3	1	2	6	Moderate
Li et al., 2018 [13]	4	2	3	9	High
Iddagoda et al., 2020 [7]	3	1	2	6	Moderate
Niu et al., 2023 [14]	4	2	3	9	High
Xiao et al., 2020 [15]	4	2	3	9	High
Chen et al., 2011 [16]	4	2	3	9	High
He et al., 2019 [17]	3	1	2	6	Moderate
Xu et al., 2023 [18]	4	2	3	9	High
Niu et al., 2023 [19]	3	1	2	6	Moderate
Tang et al., 2015 [20]	3	1	2	6	Moderate
Tsai et al., 2022 [21]	4	2	3	9	High

**Table 4:** Sleep disturbance/insomnia prevalence among stroke survivors across the included studies This table summarizes the prevalence rates of sleep disturbance or insomnia among stroke survivors reported in 13 studies. The number of patients experiencing sleep disturbance or insomnia, total sample size, calculated prevalence (%), and corresponding 95% confidence intervals (CI) are presented

Study	Sleep Disturbance/Insomnia/Total	Prevalence (%)	95% CI
Leppävuori et al., 2002 [10]	157/277	56.7%	50.8-62.5
Fan et al., 2022 [11]	1,137/1,401	81.2%	79.1-83.2
Moon et al., 2018 [12]	35/140	25.0%	17.8-32.2
Li et al., 2018 [13]	489/1,062	46.0%	43.0-49.0
Iddagoda et al., 2020 [7]	31/104	29.8%	21.0-38.6
Niu et al., 2023 [14]	89/569	15.6%	12.7-18.6
Xiao et al., 2020 [15]	76/327	23.2%	18.7-27.8
Chen et al., 2011 [16]	186/508	36.6%	32.4-40.8
He et al., 2019 [17]	24/152	15.8%	10.0-21.6
Xu et al., 2023 [18]	144/508	28.3%	24.4-32.3
Niu et al., 2023 [19]	311/530	58.7%	54.5-62.9
Tang et al., 2015 [20]	149/336	44.3%	39.0-49.7
Tsai et al., 2022 [21]	58/195	29.7%	23.3-36.2

In Table 5, the risk factor analysis, women had significantly higher odds of post-stroke insomnia than men (OR, 1.54; 95% CI, 1.25-1.88), as did unemployed individuals compared to those employed (OR, 1.41; 95% CI, 1.12-1.78). Significant associations were also found for heart conditions (OR: 1.40), anxiety (OR: 3.34), and depression (OR: 4.89), while factors such as alcohol use, smoking status, marital status, hypertension, diabetes, and hyperlipidemia were not significantly associated with post-stroke disturbance/insomnia. In Table 6, the subgroup meta-analysis revealed a higher prevalence of post-stroke sleep disturbance/insomnia in individuals under 65 years of age (54%) compared to those over 65 years (32%), though the difference was not statistically significant (p = 0.221). Regional analysis showed similar disturbance/insomnia prevalence in Asian populations and others (both 47%). By stroke type, disturbance/insomnia prevalence was nearly identical between ischemic (45%) and hemorrhagic (44%) stroke survivors. Notably, disturbance/insomnia was significantly more prevalent among patients followed for more than three months post-stroke (47%) compared to those assessed earlier (<3 months, 27%; p = 0.046). The type of sleep assessment tool used did not significantly alter prevalence estimates (objective tools, 54%; clinical criteria, 41%; self-reported, 41%; p = 0.962) nor did the categorization of symptoms as "sleep disturbance" versus "insomnia" (35% versus 41%; p = 0.669).

**Table 5:** Analysis of risk factors associated with post-stroke sleep disturbance/insomnia This table summarizes the results of random-effects meta-analyses comparing the odds of post-stroke sleep disturbance/insomnia between binary groups (e.g., female versus male and unemployed versus employed). For each comparison, the number of included studies, total events and participants per group, pooled odds ratio (OR) with 95% confidence interval (CI), and heterogeneity (I²) are reported. Odds ratios greater than 1 indicate a higher risk of sleep disturbance in Group 1 compared to Group 2. Analyses were performed using the metabin() function in the R meta package. These findings are also available in the forest plots below

Comparison	Total Studies	Group 1 (Event/Total)	Group 2 (Event/Total)	Odds Ratio (95% CI)	I² (%)
Female versus male	13	1,055/2,082	1,831/4,027	1.54 (1.25-1.88)	56
Unemployed versus employed	4	1,013/1,865	1,013/1,697	1.41 (1.12-1.78)	42
Nondrinkers versus drinkers	7	1,550/2,781	464/1,026	1.01 (0.75-1.36)	61
Nonsmokers versus smokers	8	1,465/2,968	905/1,776	1.00 (0.85-1.18)	12
Unmarried versus married	7	269/537	2,176/4,137	1.14 (0.92-1.40)	0
No hypertension versus hypertension	10	851/1,765	1,812/3,823	0.84 (0.71-1.01)	39
No diabetes versus diabetes	10	1,879/3,901	784/1,687	0.88 (0.75-1.05)	31
Heart conditions versus No heart conditions	6	88/260	614/2,183	1.40 (1.05-1.86)	0
Hyperlipidemia versus non-hyperlipidemia	5	380/794	1,767/2,859	0.99 (0.82-1.20)	0
Anxiety versus non-anxiety	3	314/395	1,056/1,610	3.34 (1.97-5.67)	57
Depression versus no depression	3	682/828	1,101/1,912	4.89 (2.48-9.68)	77

**Table 6:** Subgroup analysis for post-stroke sleep disturbance/insomnia This table summarizes pooled prevalence estimates and heterogeneity (I²) within subgroups, as well as tests for differences between subgroups using random-effects models. Pooled proportions with 95% confidence intervals (CIs) were calculated using the Freeman-Tukey double arcsine transformation. Between-subgroup differences were assessed using meta-regression or χ²-based tests, with p < 0.05 indicating statistical significance. These findings are also available in the forest plots below

Subgroup	Studies (n)	Event/Total	Pooled Proportion (95% CI)	I² (%)	Subgroup Difference (P-value)
Age					0.221
<65 years	6	2,212/4,023	0.54 (0.48-0.60)	92	
>65 years	7	671/2,086	0.32 (0.26-0.39)	90	
Region					
Asia	11	2,698/5,728	0.47 (0.42-0.52)	93	0.624
Others	2	188/381	0.47 (0.31-0.64)	69	
Stroke type					0.370
Ischemic	12	2,612/5,515	0.45 (0.40-0.50)	94	
Hemorrhagic	4	274/594	0.44 (0.34-0.54)	88	
Follow-up duration					0.046
<3 months	6	566/1,822	0.27 (0.16-0.40)	89	
>3 months	7	2,213/4,735	0.47 (0.32-0.62)	93	
Sleep disturbance assessment					0.962
Objective tools	6	1,668/3,083	0.54 (0.48-0.60)	92	
Clinical criteria	3	250/612	0.41 (0.27-0.57)	93	
Self-reported	4	968/2,414	0.41 (0.35-0.48)	92	
Definition					0.669
Disturbance	8	4,710/9,782	0.35 (0.18-0.55)	99.4	
Insomnia	5	1,399/3,259	0.41 (0.26-0.56)	96.8	

Figures 2-7 show the forest plots of subgroup analysis and factors related to post-stroke sleep disturbance/insomnia. Figures 8-12 are funnel plots evaluating publication bias for the gender-related, employment, heart condition, anxiety, and depression data.

**Figure 2:**
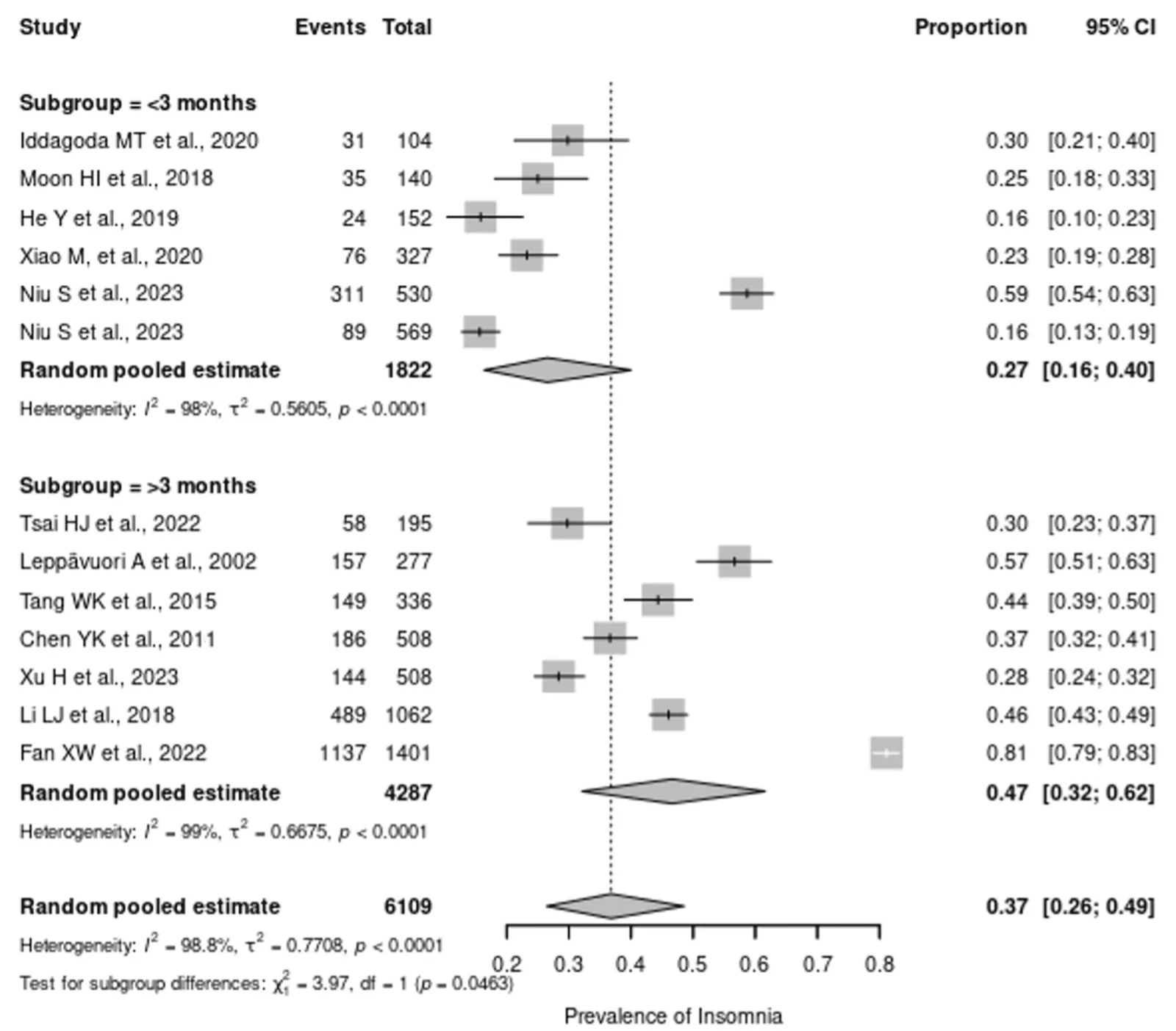
Forest plot of subgroup analysis for follow-up duration Source: [7,10-21] CI, confidence interval; df, degrees of freedom

**Figure 3:**
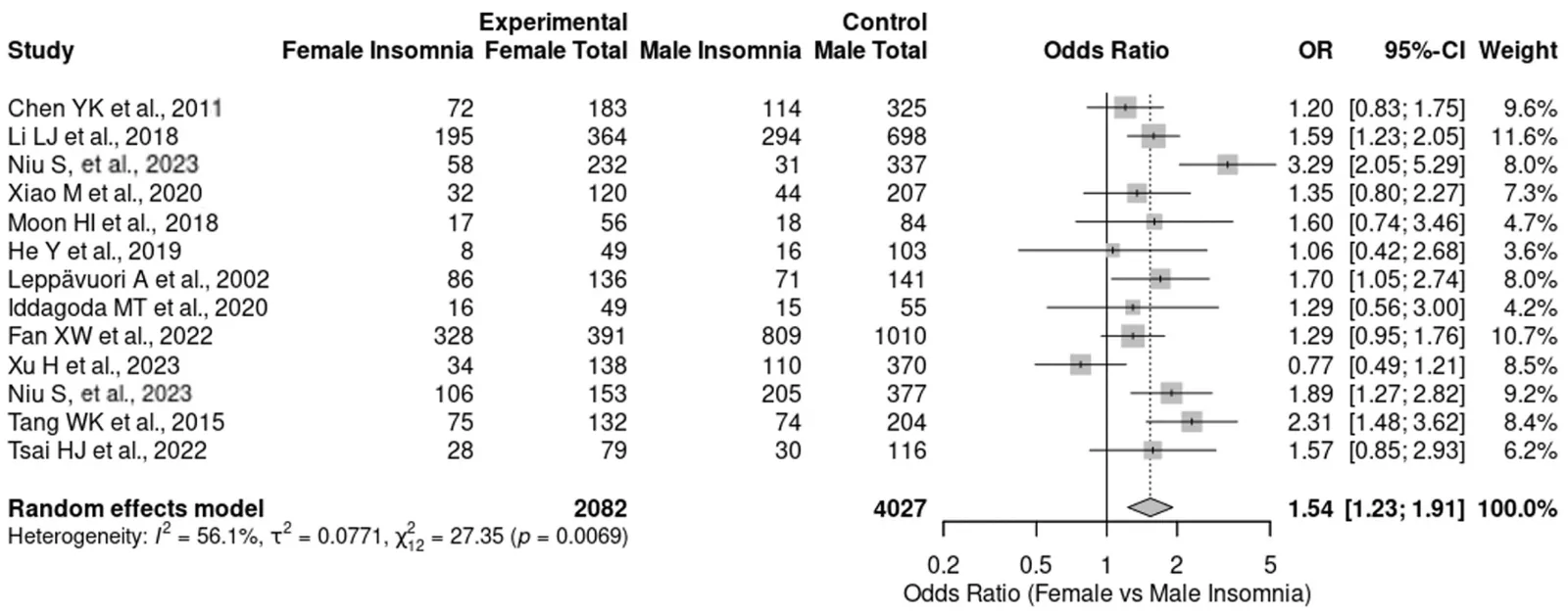
Forest plot of the gender factor associated with sleep disturbance/insomnia in post-stroke survivors Source: [7,10-21] CI: confidence interval

**Figure 4:**
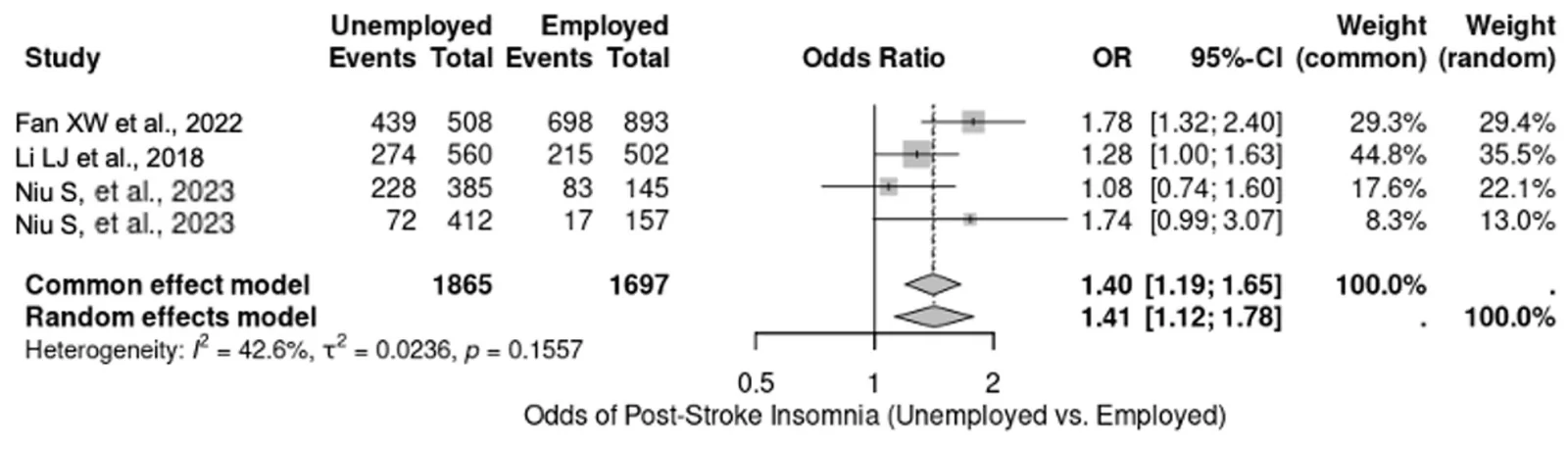
Forest plot of the employment factor associated with sleep disturbance/insomnia in post-stroke survivors Source: [11,13,14,19] CI: confidence interval

**Figure 5:**
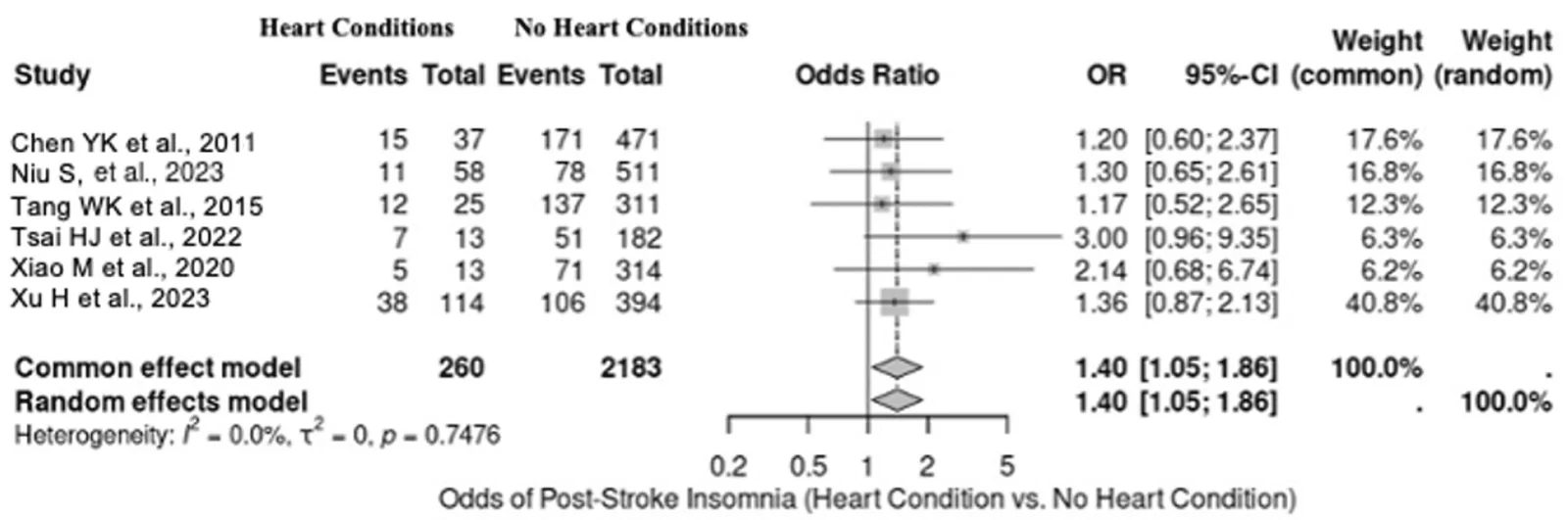
Forest plot of the heart condition factor associated with sleep disturbance/insomnia in post-stroke survivors Source: [15,16,18-21] CI: confidence interval

**Figure 6:**
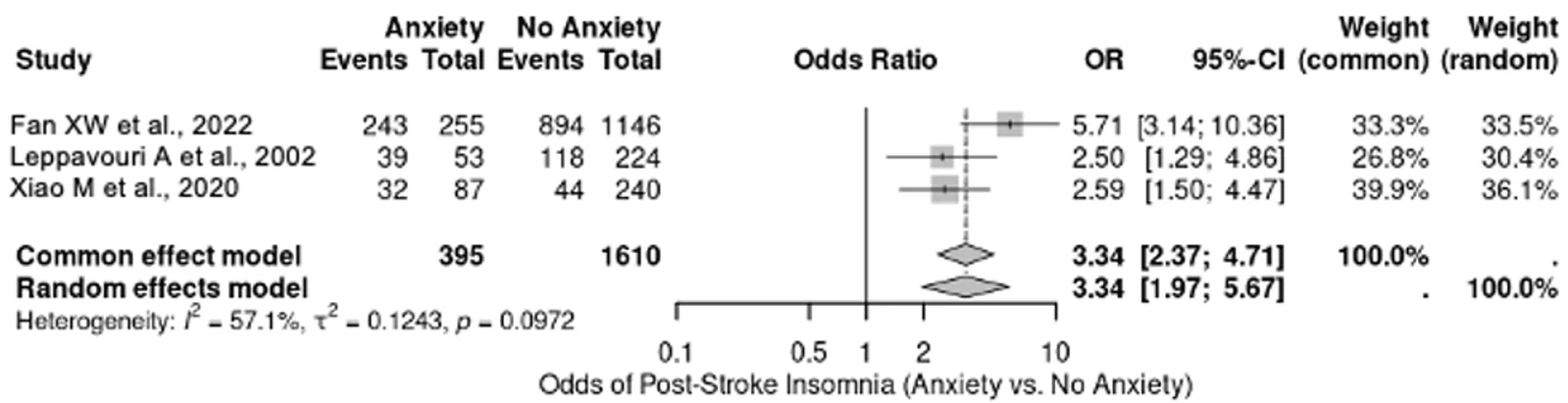
Forest plot of anxiety factor associated with sleep disturbance/insomnia in post-stroke survivors Source: [10,11,15] CI: confidence interval

**Figure 7:**
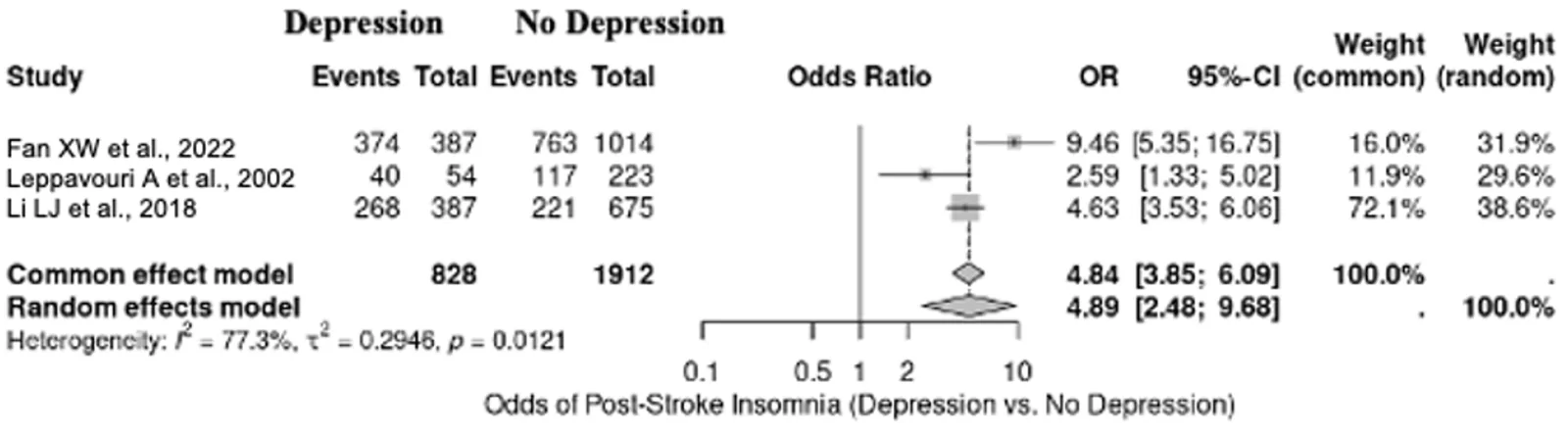
Forest plot of depression factor associated with sleep disturbance/insomnia in post-stroke survivors Source: [10,11,13] CI: confidence interval

**Figure 8:**
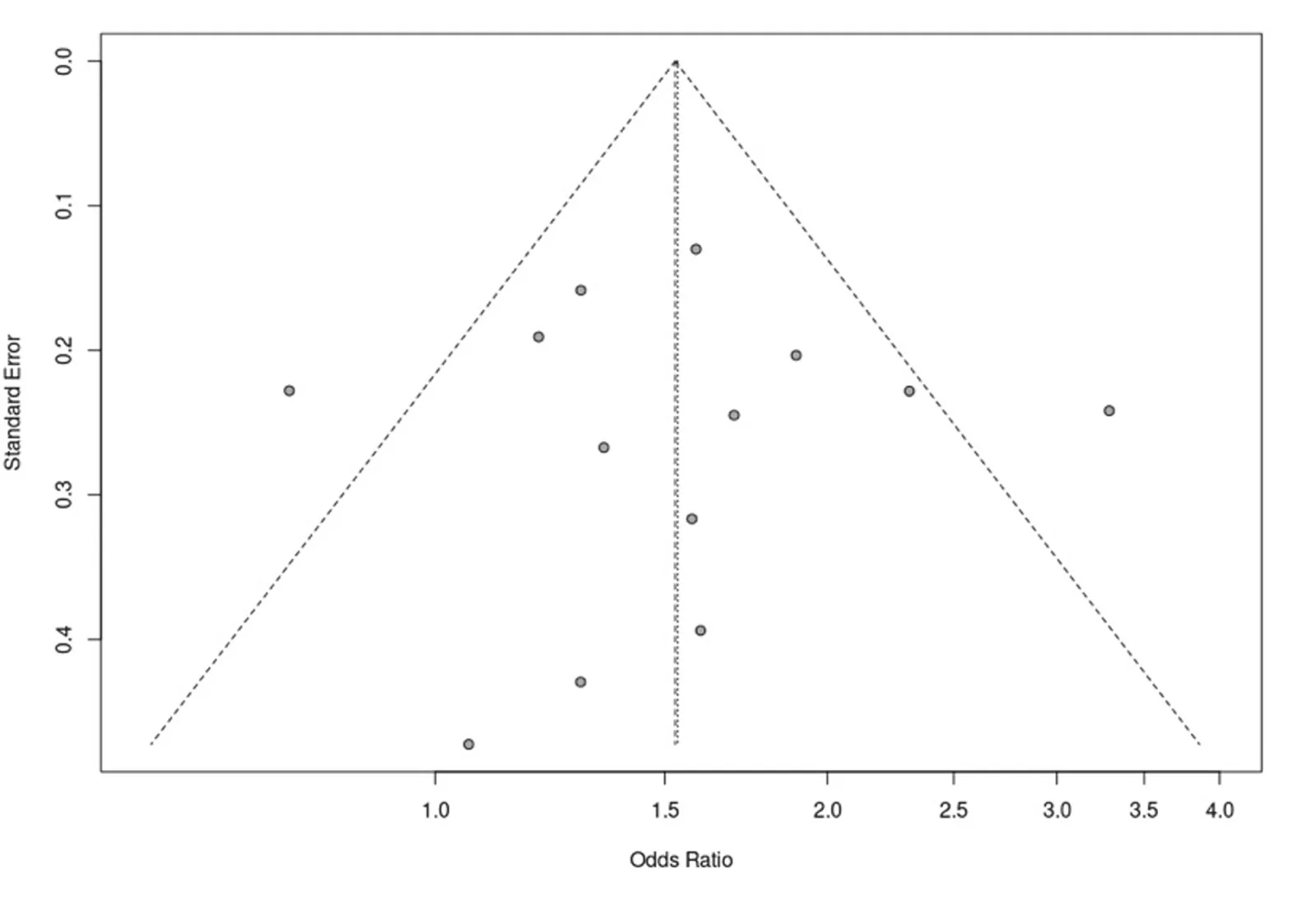
Funnel plot of bias for the gender factor associated with sleep disturbance/insomnia in post-stroke survivors

**Figure 9:**
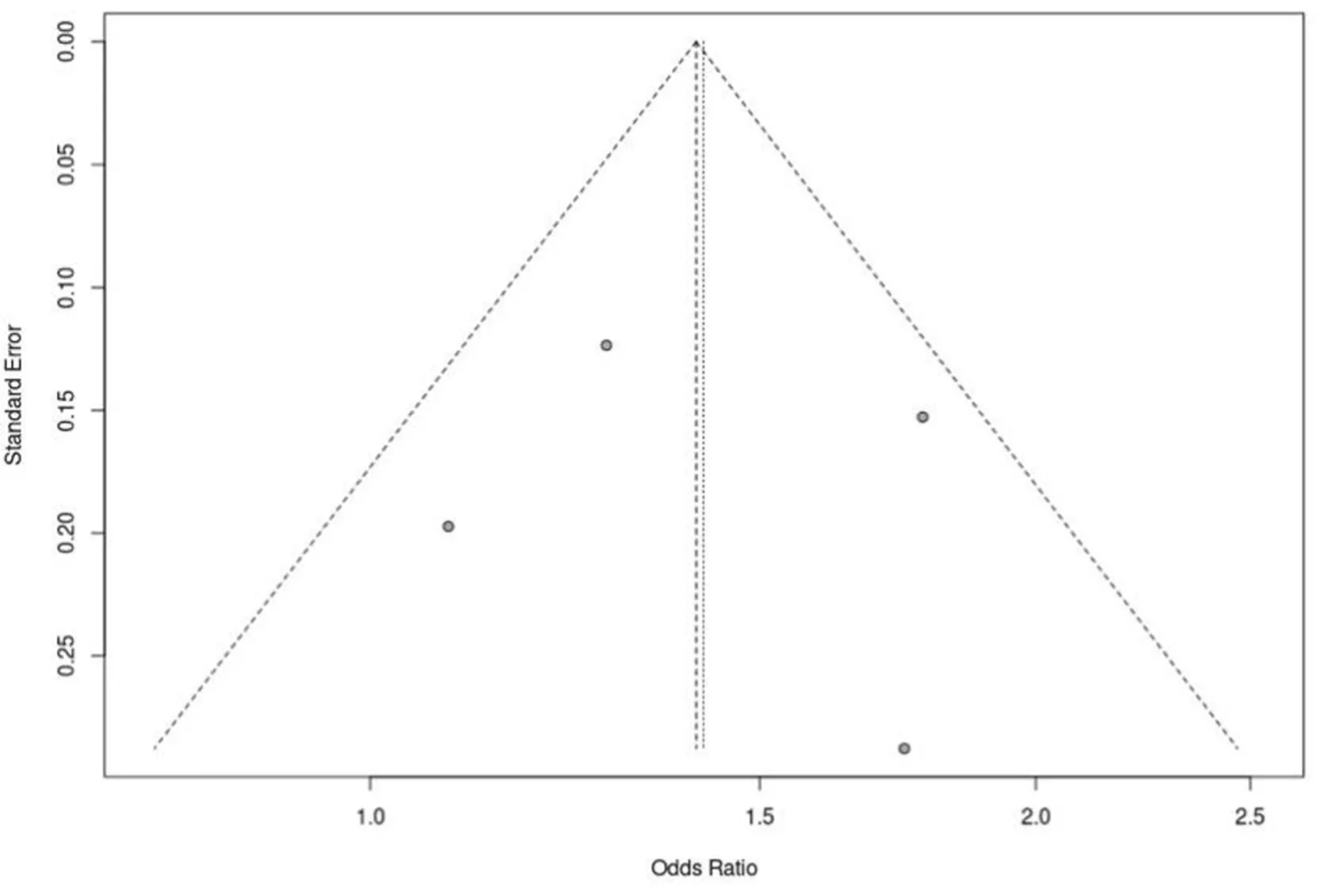
Funnel plot of bias for employment factor associated with sleep disturbance/insomnia in post-stroke survivors

**Figure 10:**
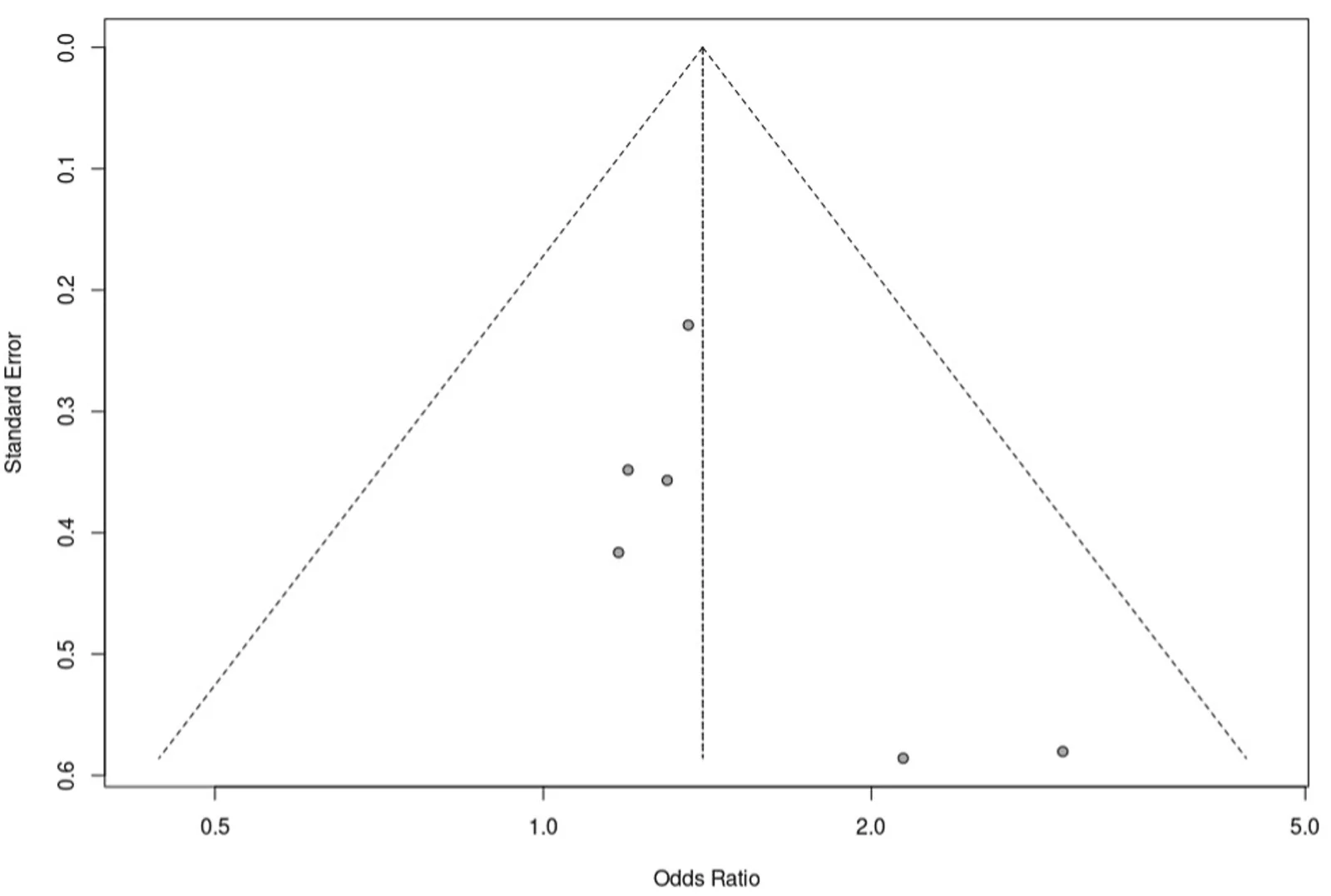
Funnel plot of bias for heart condition factor associated with sleep disturbance/insomnia in post-stroke survivors

**Figure 11:**
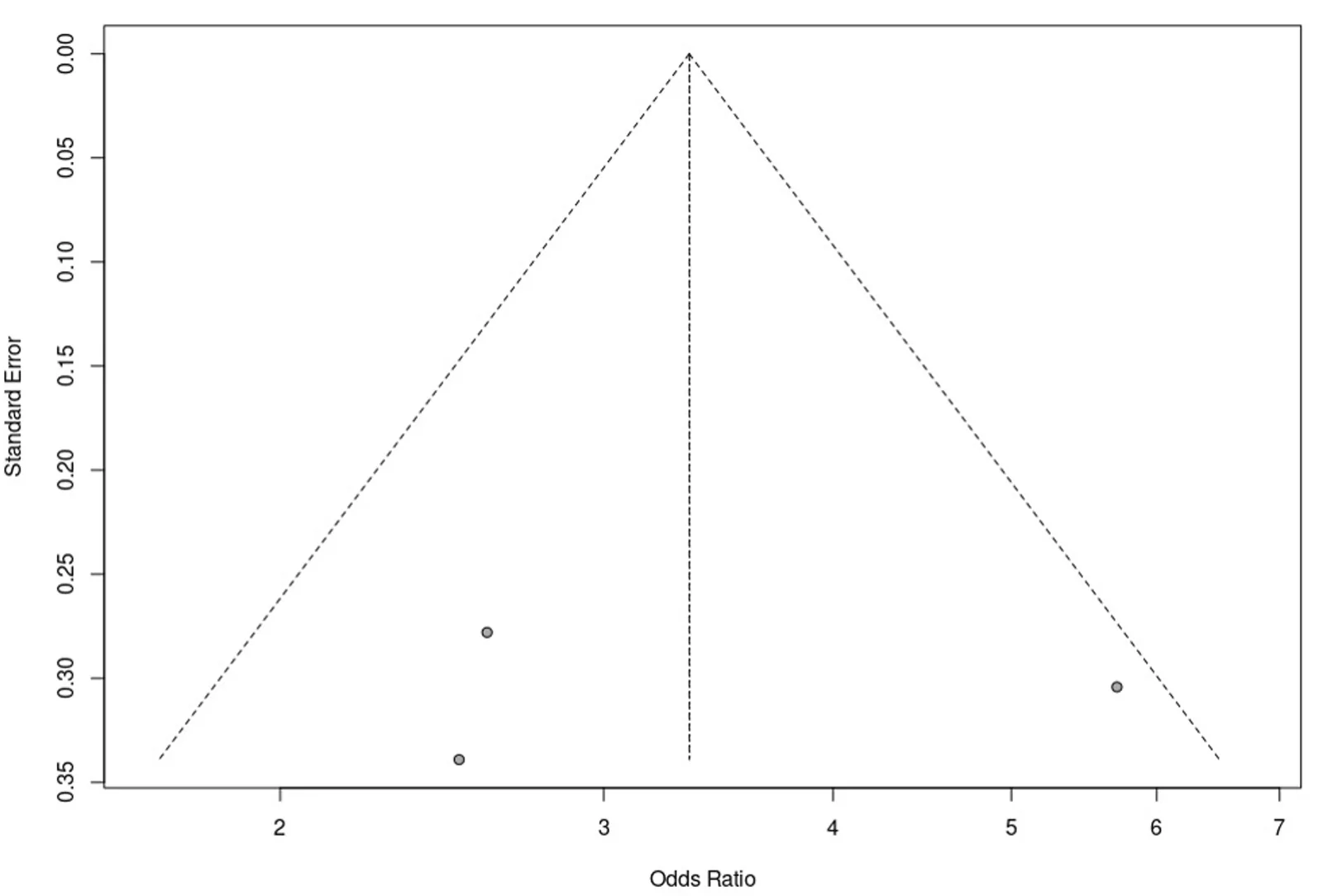
Funnel plot of bias for anxiety factor associated with sleep disturbance/insomnia in post-stroke survivors

**Figure 12:**
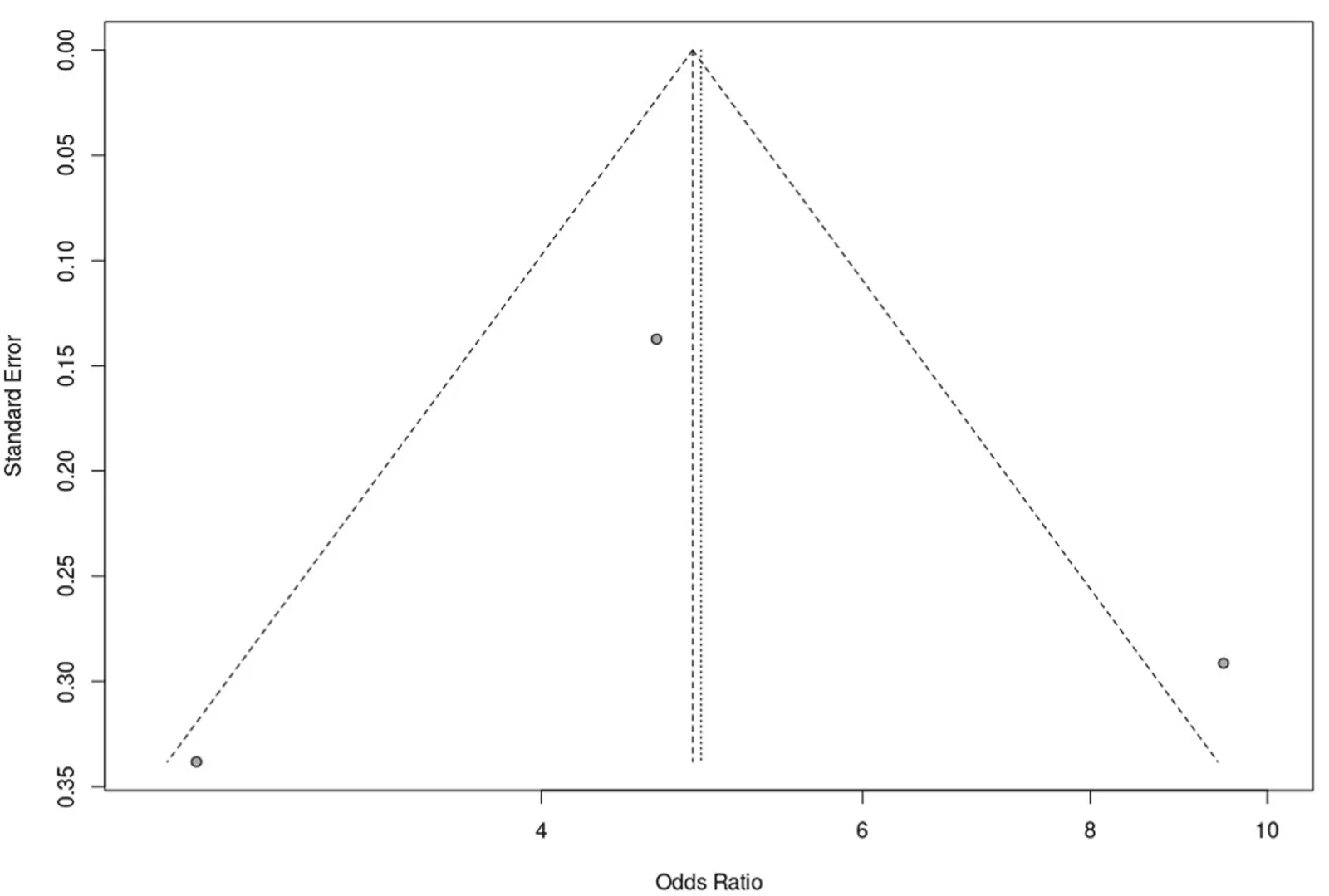
Funnel plot of bias for depression factor associated with sleep disturbance/insomnia in post-stroke survivors

The prevalence of post-stroke sleep disturbance/insomnia, calculated from extracted event and total sample data from the included studies, varied markedly across the 13 studies, ranging from 15.6% to 81.2%, highlighting the substantial heterogeneity in observed rates across different settings and methodologies. The highest prevalence was reported by Fan et al. (2022), with 81.2% of stroke survivors experiencing sleep disturbance/insomnia (95% CI: 79.1-83.2), suggesting a high burden in this cohort, which may be attributed to the large sample size, longer follow-up, or systematic use of validated sleep disturbance/insomnia measures [11]. Conversely, the lowest prevalence was observed by Niu et al. (2023) (15.6%; 95% CI, 12.7-18.6) [14] and He et al. (2019) (15.8%; 95% CI, 10.0-21.6) [17], possibly reflecting variations in diagnostic criteria, study populations, or the timing of sleep disturbance/insomnia assessment. Notably, studies with midrange prevalence estimates, such as by Leppävuori et al. (2002) (56.7%) [10], Niu et al. (2023) (58.7%) [19], and Tang et al. (2015) (44.3%) [20], may offer a more balanced estimate when accounting for methodological consistency. The wide variation in sleep disturbance and insomnia prevalence is likely influenced by several factors, including differences in assessment tools (subjective versus objective), sample characteristics (age, comorbidities, and stroke severity), the timing of evaluation, and geographical diversity.

Our subgroup analyses revealed significant differences in post-stroke sleep disturbance/insomnia only for the subgroup analysis by follow-up duration. Studies with follow-up durations longer than three months reported a higher pooled prevalence of sleep disturbance/insomnia (0.47, 0.32-0.62) compared to shorter-term studies (0.27, 0.16-0.40). This may indicate a delayed onset or persistence of sleep disturbance/insomnias as patients transition from acute to chronic recovery phases. However, the persistence of sleep disturbance/insomnia beyond three months in some patients underscores the necessity for ongoing monitoring and management strategies to address sleep disturbance/insomnia in stroke survivors. These findings emphasize the importance of the early screening and continuous assessment of sleep disorders to enhance the quality of life and functional recovery in post-stroke patients [5,22].

The analysis of post-stroke sleep disturbance/insomnia across various demographic and health-related variables reveals several significant associations that align with existing literature, providing a deeper understanding of the factors influencing sleep disturbance/insomnia in stroke survivors. The pooled analysis across 13 studies demonstrated that female stroke survivors had significantly higher odds of experiencing sleep disturbance/insomnia compared to their male counterparts (OR, 1.54; 95% CI, 1.25-1.88), with moderate heterogeneity (I² = 56%). This finding is consistent with existing literature that links the female gender with higher susceptibility to sleep disorders, potentially due to hormonal factors, the increased prevalence of mood disorders, and differential stress responses [23-25].

Similarly, unemployed individuals had a 41% higher risk of post-stroke sleep disturbance/insomnia compared to employed patients (OR, 1.41; 95% CI, 1.12-1.78). The association may reflect the psychosocial burden of joblessness, reduced daily structure, financial strain, and social isolation, all of which can disrupt circadian rhythms and sleep continuity in post-stroke populations, emphasizing the need for integrated vocational and psychological support in stroke rehabilitation programs [3].

Another clinically meaningful result emerged from the comparison between stroke survivors with and without heart disease. Individuals with pre-existing heart conditions had a 40% greater likelihood of experiencing sleep disturbance or insomnia (OR, 1.40; 95% CI, 1.05-1.86), suggesting a potential interaction between cardiovascular disease and impaired sleep regulation after stroke [26].

Psychiatric comorbidities demonstrated the most pronounced effects. Stroke survivors with anxiety had more than three times the odds of sleep disturbance/insomnia (OR, 3.34; 95% CI, 1.97-5.67), while those with depression showed nearly fivefold increased risk (OR, 4.89; 95% CI, 2.48-9.68), with high heterogeneity (I² = 77%). These findings highlight the profound interplay between mental health and sleep disturbance/insomnias following stroke and reinforce the need for integrated screening and dual-targeted intervention strategies. Addressing these comorbidities early in the rehabilitation process may improve not only sleep outcomes but also emotional regulation and functional recovery. These findings highlight the importance of routine mental health screening and the integration of evidence-based psychological interventions, such as cognitive-behavioral therapy (CBT) for sleep disturbance/insomnia and mood disorders, into post-stroke care protocols [5,11].

To address sleep disorders in stroke patients, individuals should minimize exposure to noise and light, opting for quiet environments during nighttime hours. One recommended treatment is cognitive-behavioral therapy (CBT), which has shown effectiveness in managing chronic sleep disturbance/insomnia in stroke patients requiring long-term care. However, the sustained physical and mental benefits of CBT post-stroke remain unclear and warrant further investigation with larger sample sizes in future studies [27,28].

Limitations and research gap

While this study provides valuable insights into the prevalence and factors associated with post-stroke sleep disturbance and insomnia, several limitations should be considered. First, substantial heterogeneity was observed across several analyses, likely reflecting differences in study populations, sleep disturbance/insomnia definitions, assessment methods, follow-up duration, and study designs. Subgroup analyses were performed to explore potential sources of heterogeneity; however, considerable variability remained across several subgroup analyses, which may affect the generalizability of the findings. Second, the reliance on self-reported data for certain variables, such as sleep disturbance or insomnia diagnosis, may introduce biases and affect the accuracy of the prevalence estimates. Third, the use of odds ratios in studies with relatively common outcomes may overestimate the strength of associations. Fourth, some full-text studies could not be retrieved due to access limitations, and author contact for unavailable studies was not performed, which may have introduced selection bias. Fifth, the studies included in this meta-analysis primarily focus on specific regions, particularly in Asian countries, which limits the global applicability of the results. Furthermore, the lack of longitudinal studies and the inability to explore the impact of treatment interventions on sleep disturbance/insomnia over time leave a gap in understanding the long-term management of post-stroke sleep disturbance/insomnia. Future research should focus on conducting large-scale, multicenter, and longitudinal studies to address these gaps and develop standardized assessment tools for sleep disturbance/insomnia in stroke populations across diverse geographical and clinical settings. In addition, incorporating sleep hygiene education into stroke rehabilitation and follow-up care may help improve sleep outcomes and overall recovery in stroke survivors.

## Conclusions

This study highlights that post-stroke sleep disturbance/insomnia is common among stroke survivors, particularly among women, unemployed individuals, and those with depression, anxiety, or heart disease. Depression and anxiety showed the strongest associations with sleep disturbance/insomnia. Despite substantial heterogeneity between studies, the findings support the importance of routine screening using validated assessment tools and multidisciplinary care addressing both sleep and mental health in stroke survivors. Early recognition and intervention may help improve recovery and the quality of life. Further research is needed to evaluate the impact of pre-existing sleep disorders on post-stroke sleep outcomes.

## Figures and Tables

**Figure 13:**
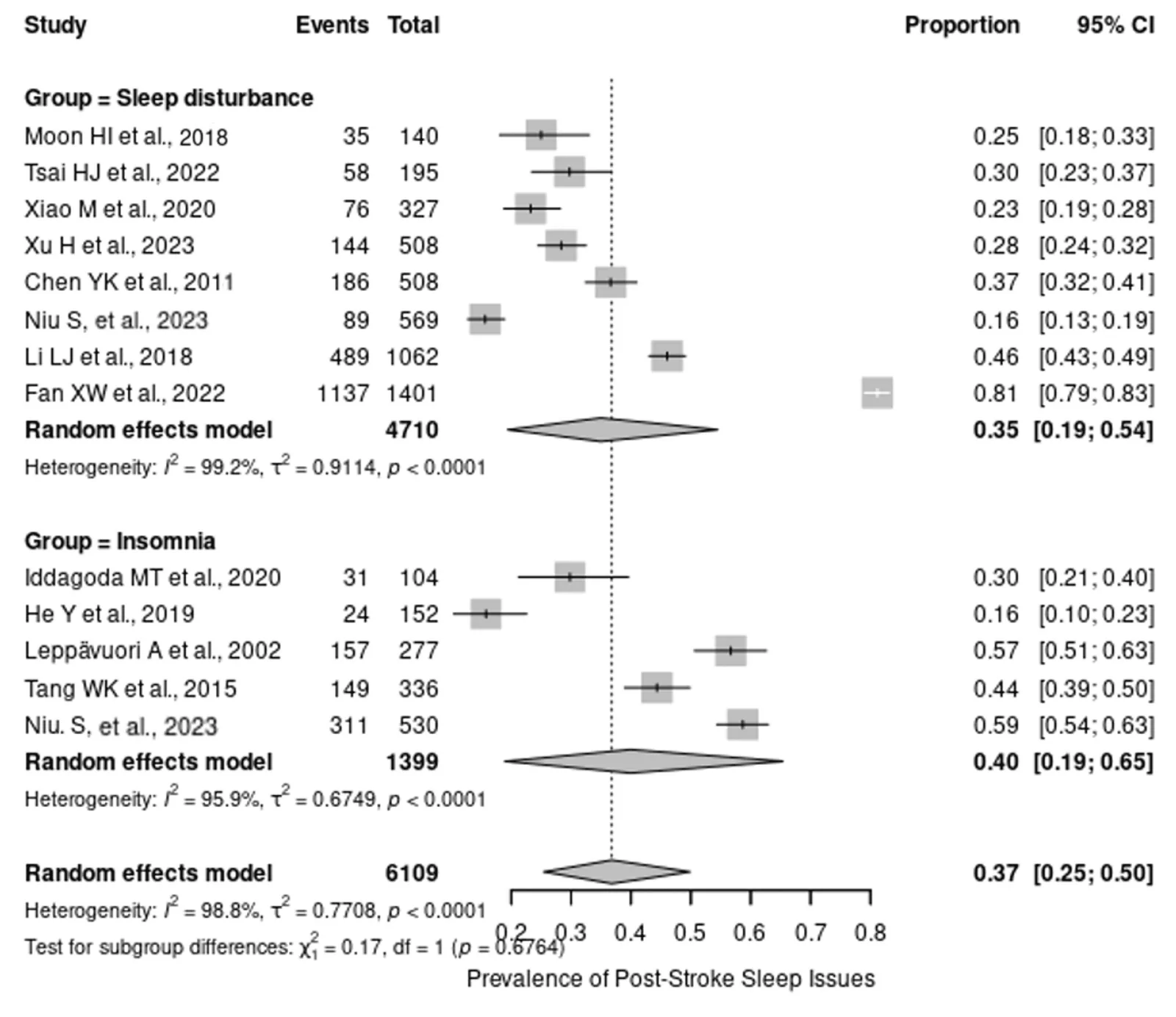
Forest plot of subgroup analysis between sleep disturbance and insomnia Source: [7,10-21] CI, confidence interval; df, degrees of freedom

**Figure 14:**
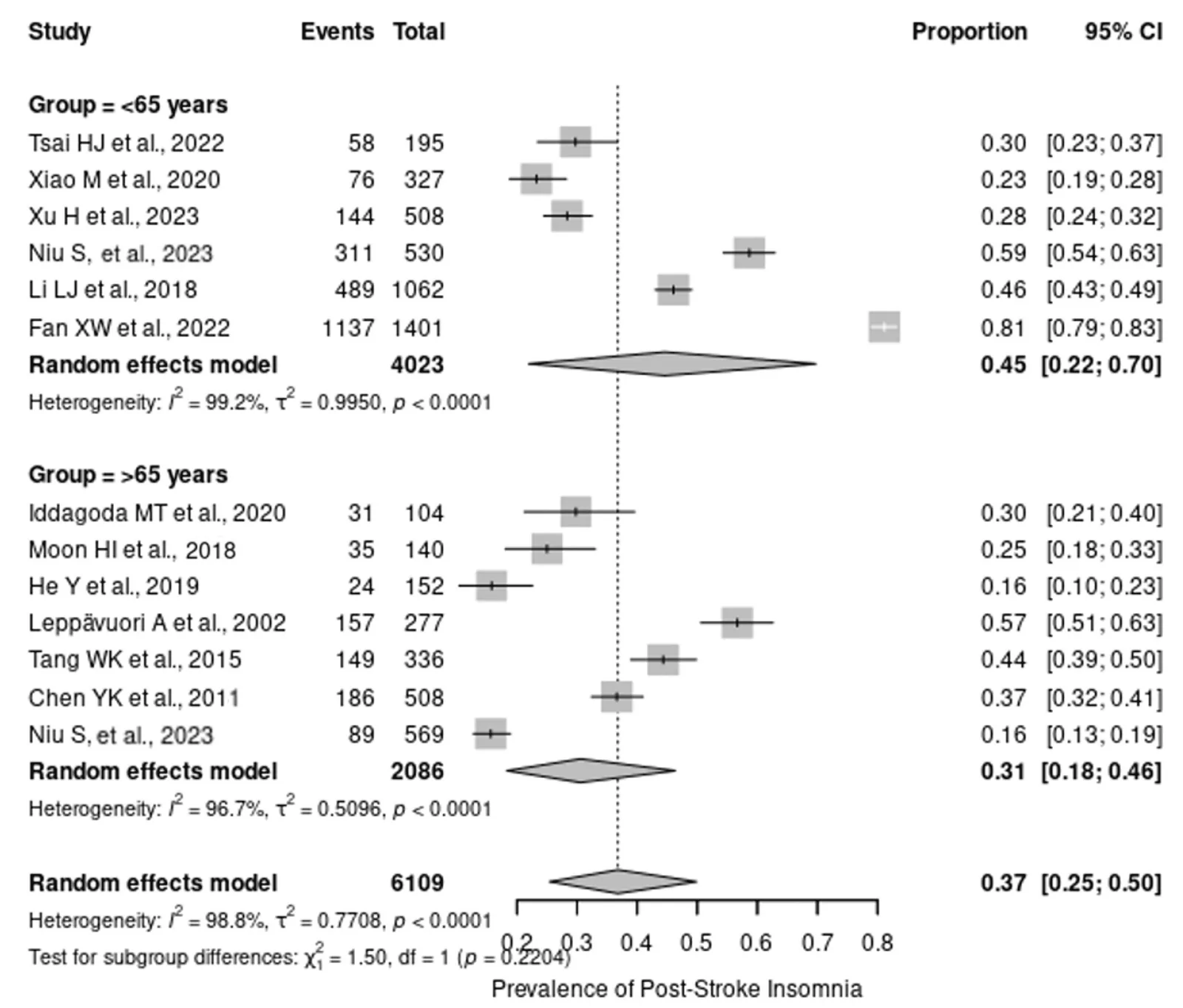
Forest plot of subgroup analysis by age groups Source: [7,10-21] CI, confidence interval; df, degrees of freedom

**Figure 15:**
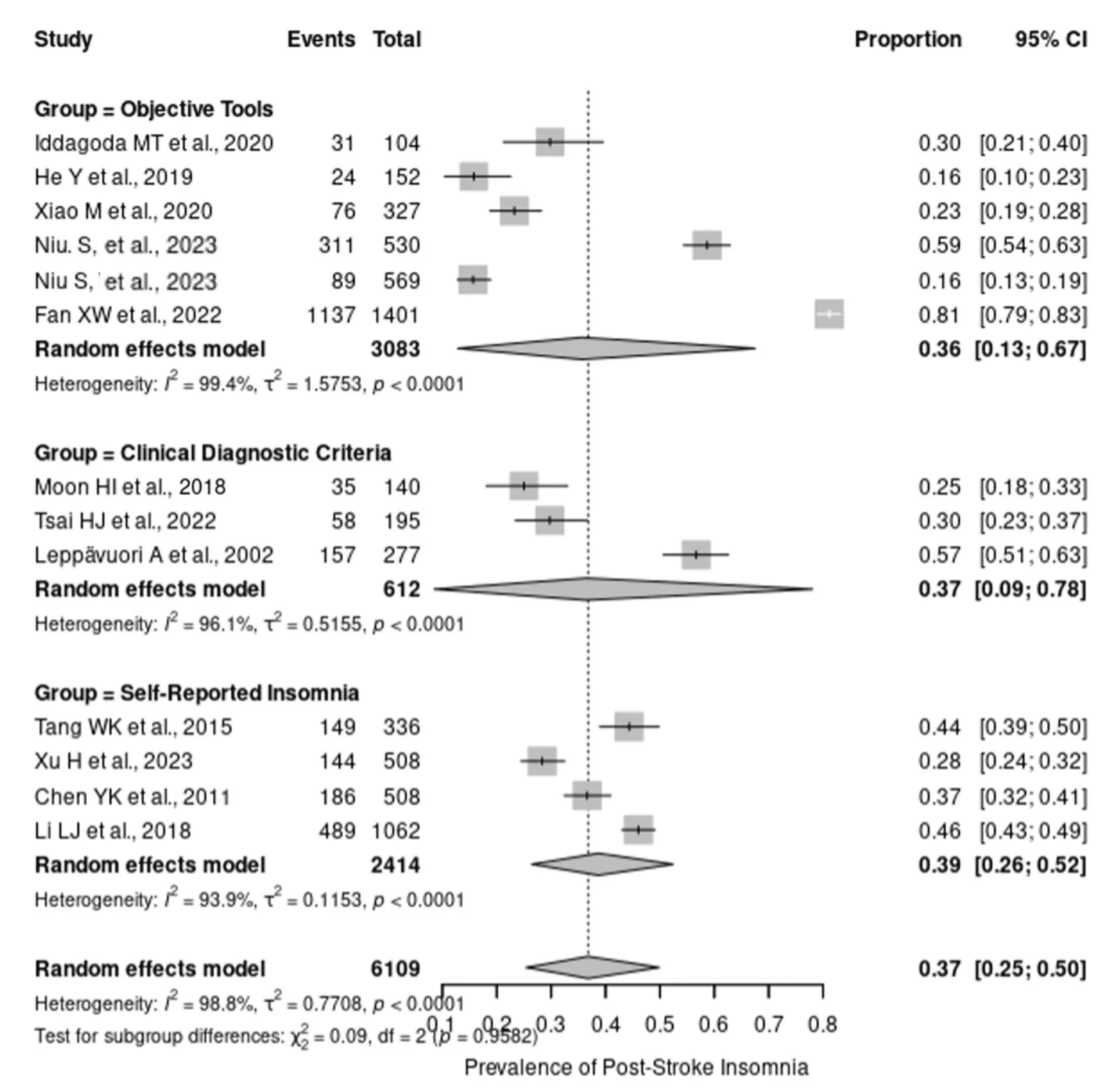
Forest plot of subgroup analysis for definition type Source: [7,10-21] CI, confidence interval; df, degrees of freedom

**Figure 16:**
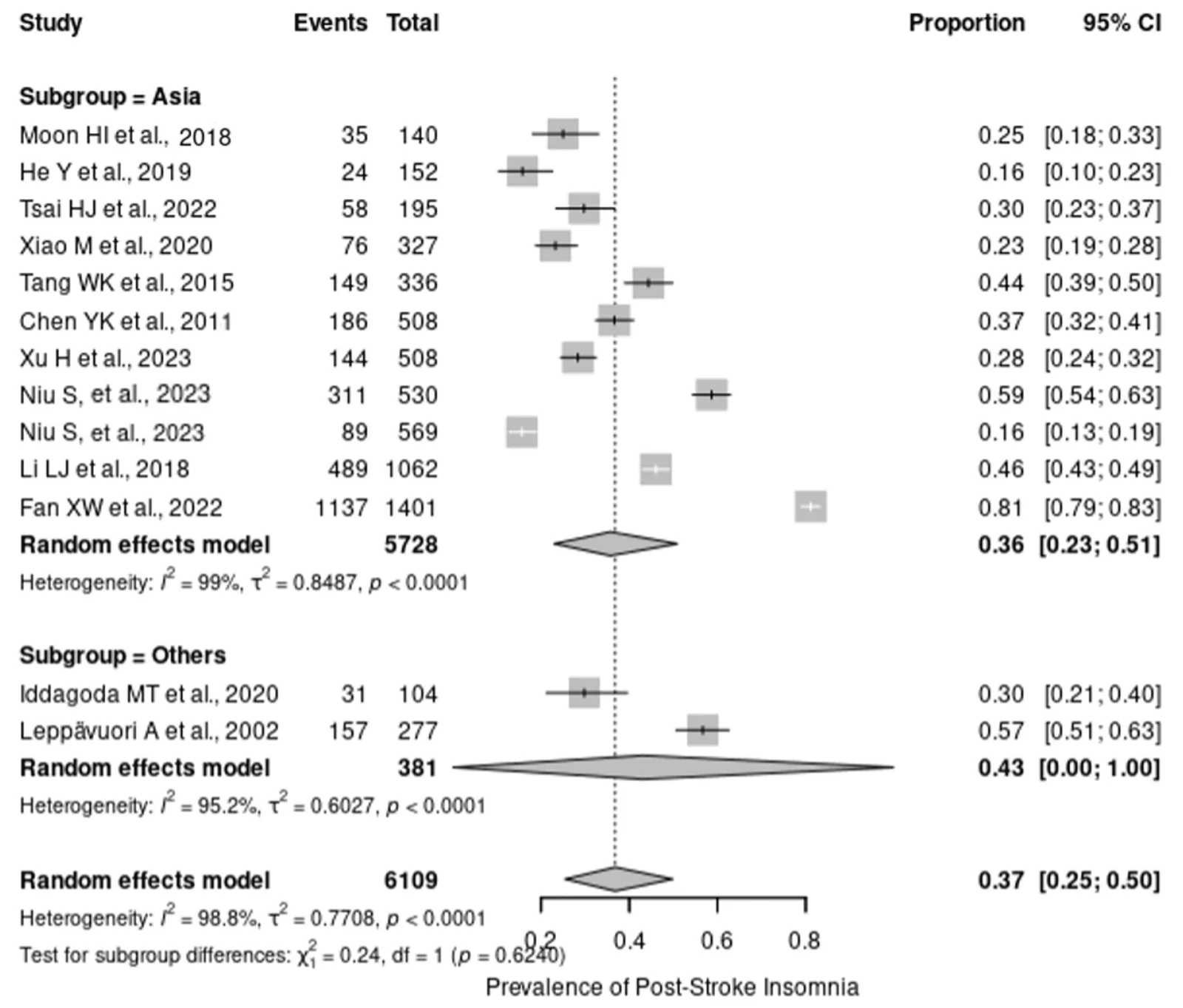
Forest plot of subgroup analysis by region Source: [7,10-21] CI, confidence interval; df, degrees of freedom

**Figure 17:**
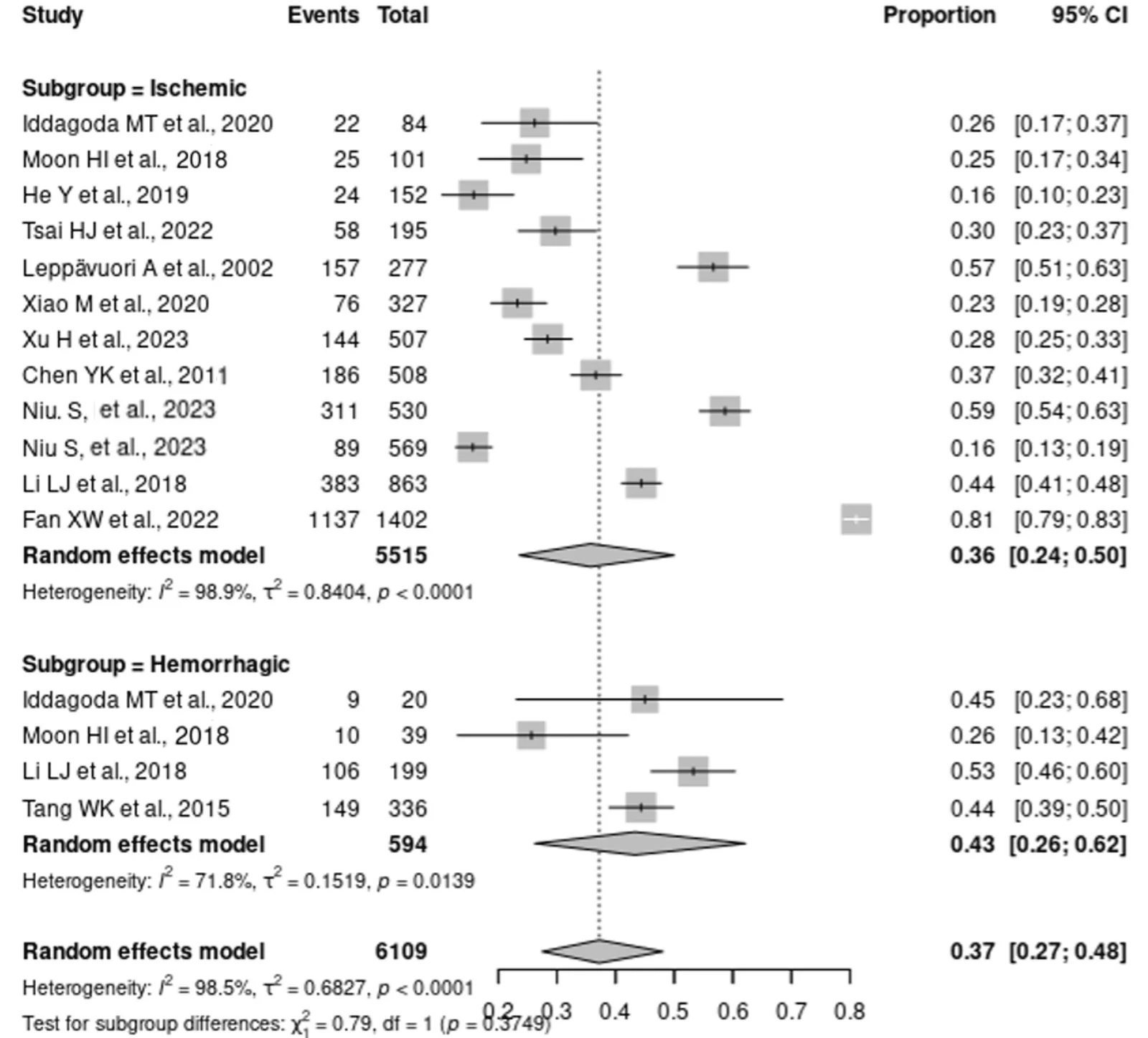
Forest plot of subgroup analysis by stroke type Source: [7,10-21] CI, confidence interval; df, degrees of freedom

## Appendices

Figures 13-17 also show forest plots of subgroup analysis and factors related to post-stroke sleep disturbance/insomnia.
